# Supplementary material for: Comparative single-cell and spatial profiling of anti-SSA-positive and anti-centromere-positive Sjögren’s disease reveals common and distinct immune activation and fibroblast-mediated inflammation
Source: Nat Commun. 2025 Sep 22;16:8299. doi: 10.1038/s41467-025-63935-9 (PMC12454658; doi:10.1038/s41467-025-63935-9)
Supplement: Supplementary file 1 — Supplementary Information [file 41467_2025_63935_MOESM1_ESM.pdf]

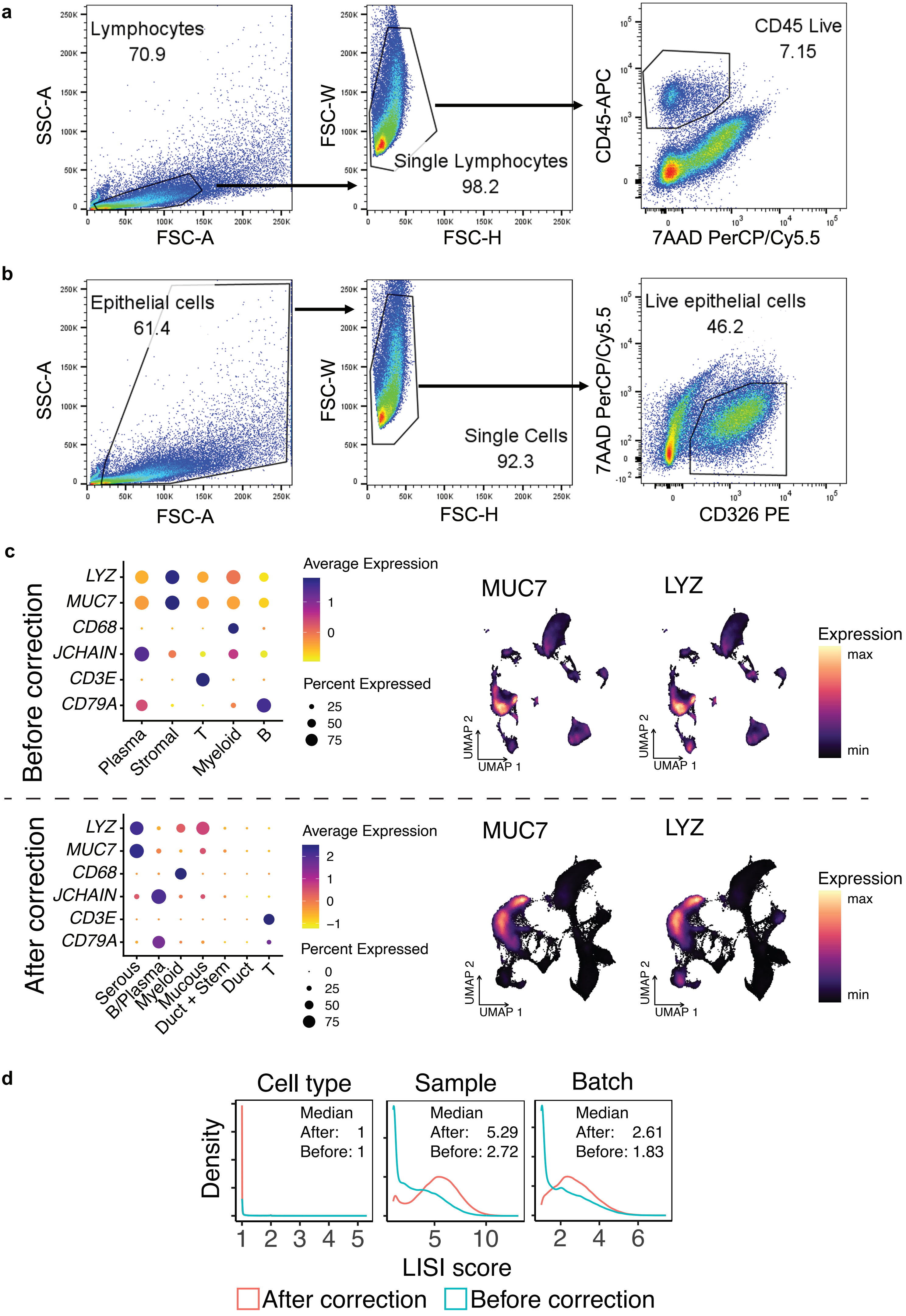

a
Lymphocytes
70.9
SSC-A
FSC-A
Single Lymphocytes
98.2
FSC-W
FSC-H
CD45 Live
7.15
CD45-APC
7AAD PerCP/Cy5.5
b
Epithelial cells
61.4
SSC-A
FSC-A
Single Cells
92.3
FSC-W
FSC-H
Live epithelial cells
46.2
7AAD PerCP/Cy5.5
CD326 PE
c
Before correction
LYZ
MUC7
CD68
JCHAIN
CD3E
CD79A
Plasma
Stromal
T
Myeloid
B
Average Expression
Percent Expressed
25
50
75
MUC7
LYZ
UMAP 2
UMAP 1
Expression
max
min
After correction
Serous
B/Plasma
Myeloid
Mucous
Duct + Stem
Duct
T
Average Expression
Percent Expressed
0
25
50
75
d
Cell type
Median
After: 1
Before: 1
Sample
Median
After: 5.29
Before: 2.72
Batch
Median
After: 2.61
Before: 1.83
Density
LISI score
After correction
Before correction

Supplementary Figure 1: Correction of background noise and batch effect improves data quality and mixture levels in single-cell transcriptome analysis.

a-b, Gating strategies for (**a**) lymphocyte and (**b**) salivary gland epithelial cells. **c,** Impact of background noise correction on gene expression profiles. Dot plot displays the percentage of cells expressing selected genes before (top) and after (bottom) correction using CellBender. UMAP plots present the gene expression of *MUC7* and *LYZ* on UMAP coordinates before and after correction as representative examples, with the color intensity indicating the expression level from min to max. **d**, Evaluation of mixture levels using LISI (Local Inverse Simpson's Index) scores before and after batch effect correction using Harmony. The density plots display the distribution of LISI scores for cell type, sample, and batch categories. The median LISI scores before and after correction are shown for each category. Higher LISI scores after correction indicate improved mixture levels and reduced batch effects.

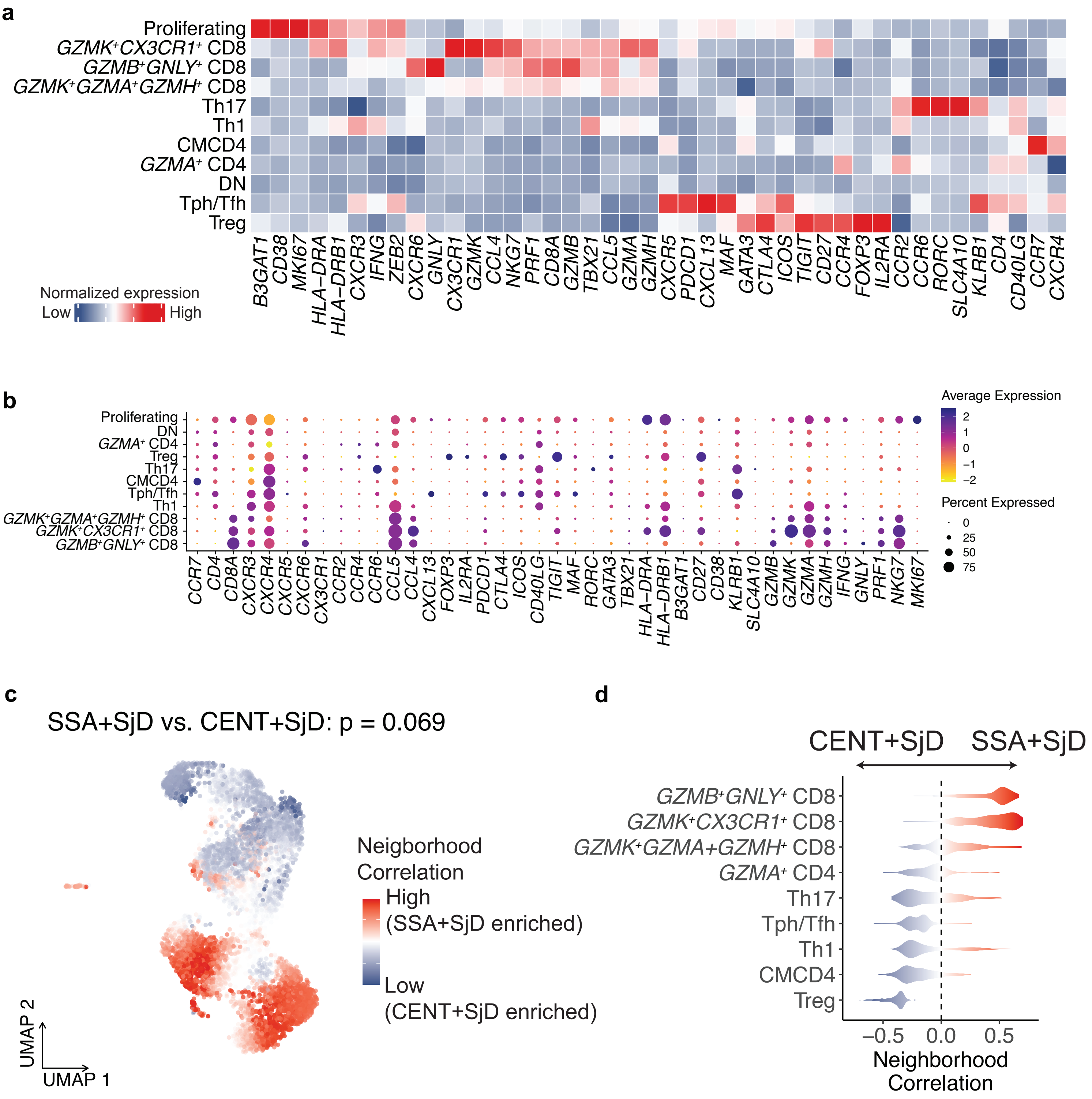
a
Proliferating
GZMK+CX3CR1+ CD8
GZMB+GNLY+ CD8
GZMK+GZMA+GZMH+ CD8
Th17
Th1
CMCD4
GZMA+ CD4
DN
Tph/Tfh
Treg
B3GAT1 CD38 MKI67 HLA−DRA HLA−DRB1 CXCR3 IFNG ZEB2 CXCR6 GNLY CX3CR1 GZMK CCL4 NKG7 PRF1 CD8A GZMB TBX21 CCL5 GZMA GZMH CXCR5 PDCD1 CXCL13 MAF GATA3 CTLA4 ICOS TIGIT CD27 CCR4 FOXP3 IL2RA CCR2 CCR6 RORC SLC4A10 KLRB1 CD4 CD40LG CCR7 CXCR4
Normalized expression
Low
High
b
Proliferating
DN
GZMA+ CD4
Treg
Th17
CMCD4
Tph/Tfh
Th1
GZMK+GZMA+GZMH+ CD8
GZMK+CX3CR1+ CD8
GZMB+GNLY+ CD8
CCR7 CD4 CD8A CXCR3 CXCR4 CXCR5 CXCR6 CX3CR1 CCR2 CCR4 CCR6 CCL5 CCL4 CXCL13 FOXP3 IL2RA PDCD1 CTLA4 ICOS CD40LG TIGIT MAF RORC GATA3 TBX21 HLA−DRA HLA−DRB1 B3GAT1 CD27 CD38 KLRB1 SLC4A10 GZMB GZMK GZMA GZMH IFNG GNLY PRF1 NKG7 MKI67
Average Expression
2
1
0
−1
−2
Percent Expressed
0
25
50
75
c
SSA+SjD vs. CENT+SjD: p = 0.069
Neigborhood Correlation
High (SSA+SjD enriched)
Low (CENT+SjD enriched)
UMAP 2
UMAP 1
d
CENT+SjD
SSA+SjD
GZMB+GNLY+ CD8
GZMK+CX3CR1+ CD8
GZMK+GZMA+GZMH+ CD8
GZMA+ CD4
Th17
Tph/Tfh
Th1
CMCD4
Treg
−0.5
0.0
0.5
Neighborhood Correlation

Supplementary Figure 2: Expression of key markers in T cell subsets.
a-b, Heatmap (**a**) and dot plot (**b**) showing expression of marker genes. In heatmap, column-wise Z scores of normalized expressions are plotted. **c**, Differential abundance analysis comparing SSA+ SjD and CENT+ SjD by co-varying neighborhood analysis. Cells in UMAP are colored in red (expansion) or blue (depletion) and p-value is shown as well. **d**, Distributions of cell neighborhood correlations by T cell clusters.

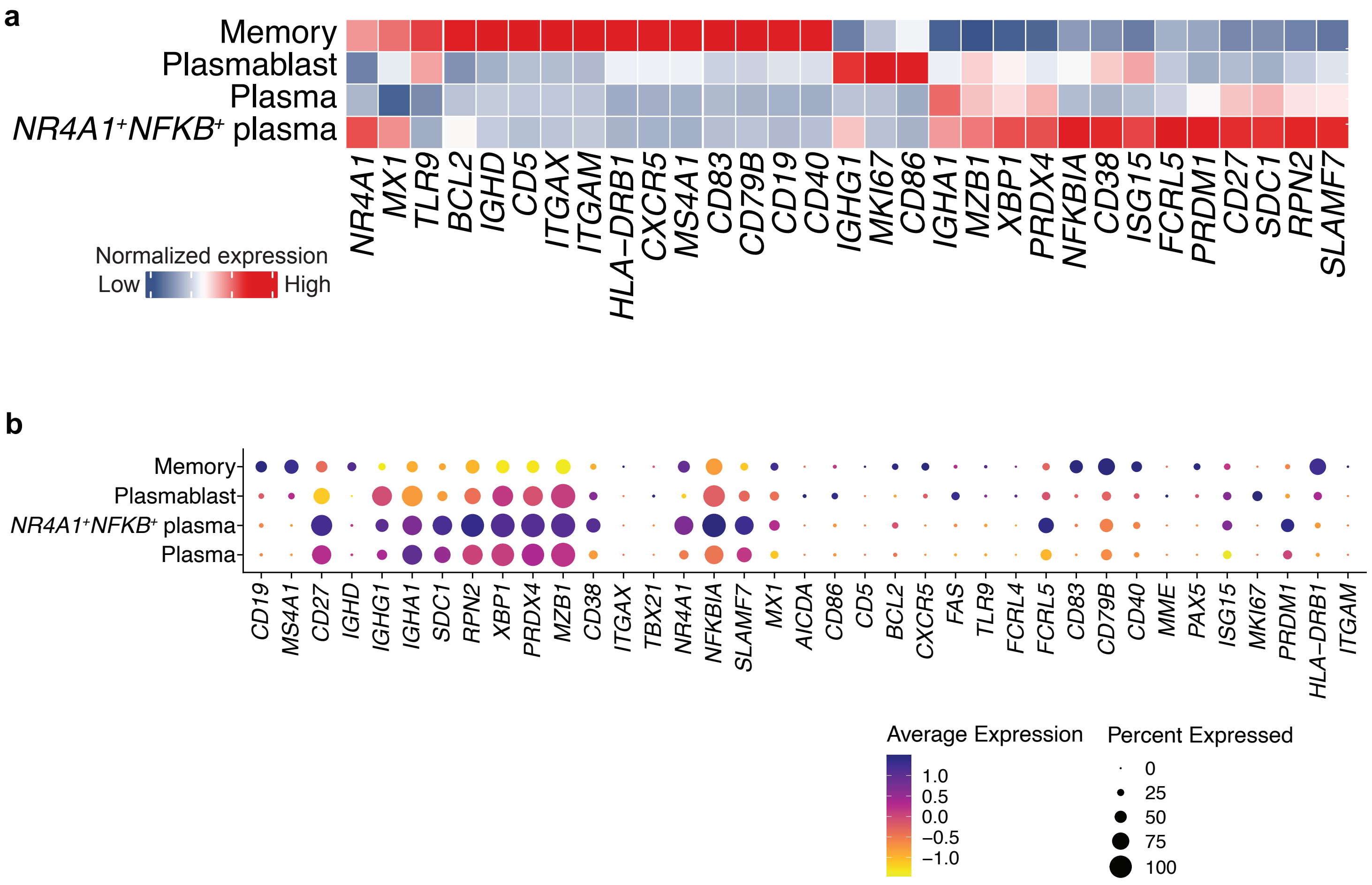

a
Memory
Plasmablast
Plasma
NR4A1+NFKB+ plasma
NR4A1 MX1 TLR9 BCL2 IGHD CD5 ITGAX ITGAM HLA-DRB1 CXCR5 MS4A1 CD83 CD79B CD19 CD40 IGHG1 MKI67 CD86 IGHA1 MZB1 XBP1 PRDX4 NFKBIA CD38 ISG15 FCRL5 PRDM1 CD27 SDC1 RPN2 SLAMF7
Normalized expression
Low
High
b
Memory
Plasmablast
NR4A1+NFKB+ plasma
Plasma
CD19 MS4A1 CD27 IGHD IGHG1 IGHA1 SDC1 RPN2 XBP1 PRDX4 MZB1 CD38 ITGAX TBX21 NR4A1 NFKBIA SLAMF7 MX1 AICDA CD86 CD5 BCL2 CXCR5 FAS TLR9 FCRL4 FCRL5 CD83 CD79B CD40 MME PAX5 ISG15 MKI67 PRDM1 HLA-DRB1 ITGAM
Average Expression
1.0
0.5
0.0
−0.5
−1.0
Percent Expressed
0
25
50
75
100

Supplementary Figure 3: Expression of key markers in B/plasma cell subsets.
a-b, Heatmap (**a**) and dot plot (**b**) showing expression of marker genes. In heatmap, column-wise Z scores of normalized expressions are plotted.

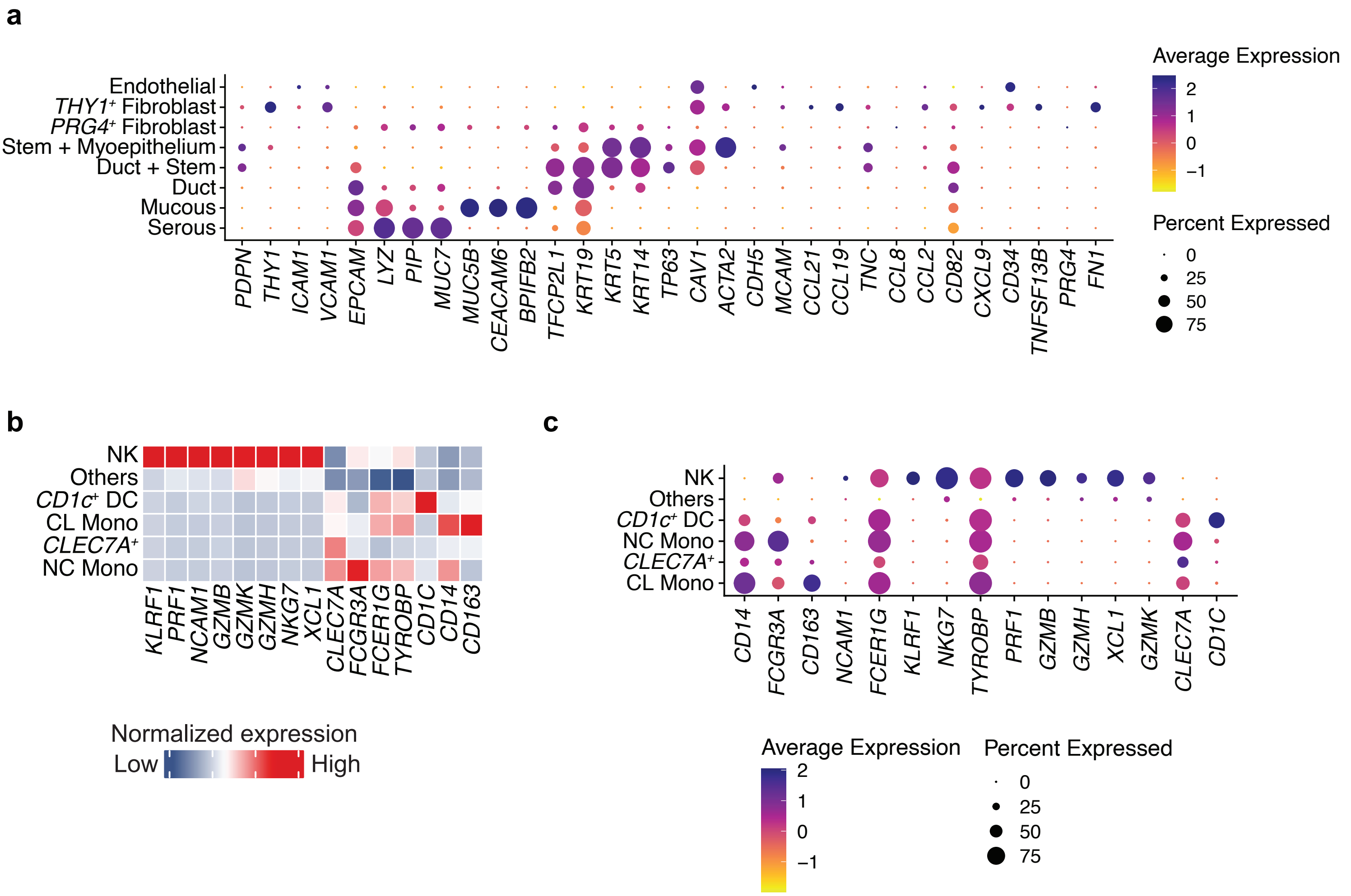
a
Endothelial
THY1+ Fibroblast
PRG4+ Fibroblast
Stem + Myoepithelium
Duct + Stem
Duct
Mucous
Serous
PDPN
THY1
ICAM1
VCAM1
EPCAM
LYZ
PIP
MUC7
MUC5B
CEACAM6
BPIFB2
TFCP2L1
KRT19
KRT5
KRT14
TP63
CAV1
ACTA2
CDH5
MCAM
CCL21
CCL19
TNC
CCL8
CCL2
CD82
CXCL9
CD34
TNFSF13B
PRG4
FN1
Average Expression
2
1
0
−1
Percent Expressed
0
25
50
75
b
NK
Others
CD1c+ DC
CL Mono
CLEC7A+
NC Mono
KLRF1
PRF1
NCAM1
GZMB
GZMK
GZMH
NKG7
XCL1
CLEC7A
FCGR3A
FCER1G
TYROBP
CD1C
CD14
CD163
Normalized expression
Low
High
c
NK
Others
CD1c+ DC
NC Mono
CLEC7A+
CL Mono
CD14
FCGR3A
CD163
NCAM1
FCER1G
KLRF1
NKG7
TYROBP
PRF1
GZMB
GZMH
XCL1
GZMK
CLEC7A
CD1C
Average Expression
2
1
0
−1
Percent Expressed
0
25
50
75

Supplementary Figure 4: Expression of key markers in tissue cell and other leukocyte subsets.

a, Dot plot showing expression of marker genes in tissue cell subsets. **b-c**, Heatmap (**b**) and dot plot (**c**) showing expression of marker genes in other leukocyte subsets. In heatmap, column-wise Z scores of normalized expressions are plotted.

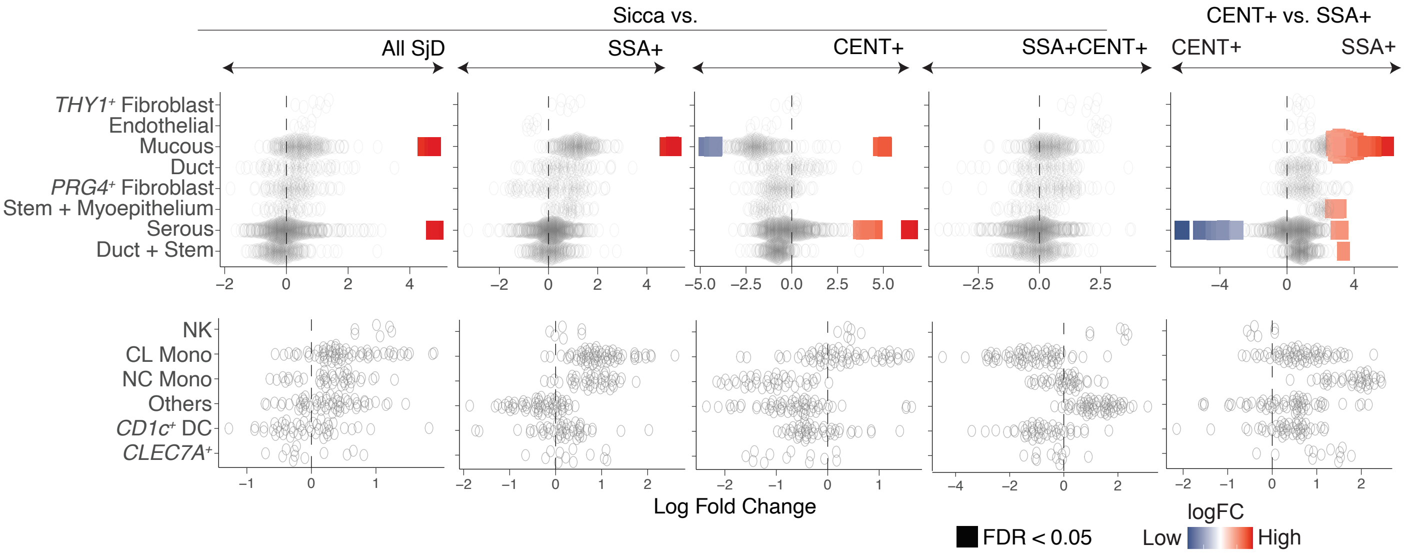

Sicca vs.
CENT+ vs. SSA+
All SjD
SSA+
CENT+
SSA+CENT+
CENT+
SSA+
THY1+ Fibroblast
Endothelial
Mucous
Duct
PRG4+ Fibroblast
Stem + Myoepithelium
Serous
Duct + Stem
NK
CL Mono
NC Mono
Others
CD1c+ DC
CLEC7A+
Log Fold Change
logFC
FDR < 0.05
Low
High

Supplementary Figure 5: Differential abundance profiles of tissue and other leukocyte cell populations in SjD subtypes.

Beeswarm plots showing the distribution of log_2-fold change (logFC) in neighborhoods in different cell type clusters in tissue cells (top), and myeloid and NK cells (bottom). Each plot compares the abundance in SjD overall, SSA+, CENT+, and SSA+CENT+ subtypes versus Sicca or each other. Significant changes (FDR < 0.05) are highlighted, indicating enriched or depleted in each case.

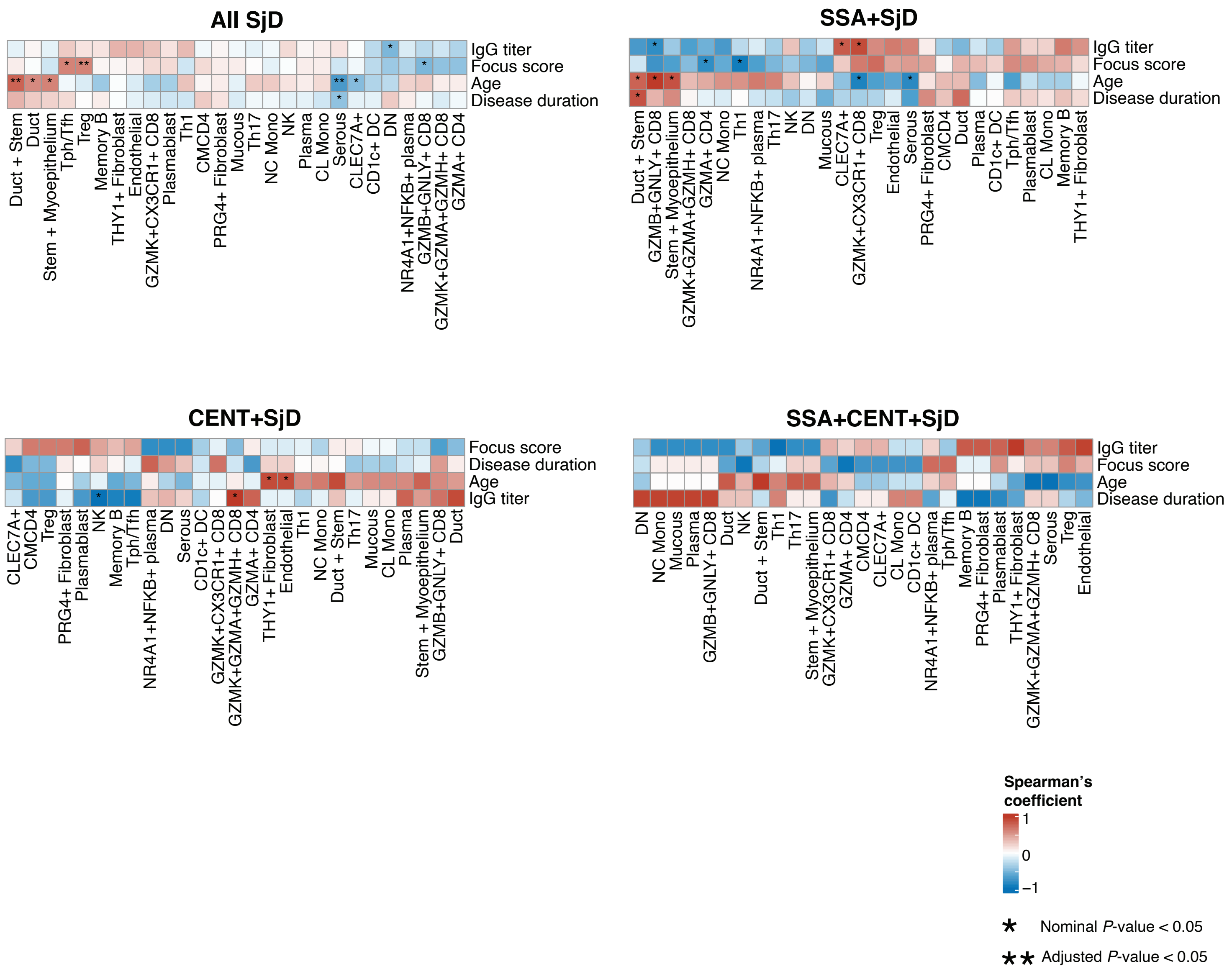

All SjD
SSA+SjD
CENT+SjD
SSA+CENT+SjD
IgG titer
Focus score
Age
Disease duration
Spearman's coefficient
1
0
−1
* Nominal P-value < 0.05
** Adjusted P-value < 0.05

Supplementary Figure 6: Correlation between cell-type frequencies and clinical features. Heatmaps showing Spearman's correlations between fine-grained immune and stromal cell clusters and clinical variables. T, B/plasma, and other leukocyte subsets are normalized within their respective parent lineages, while tissue cells are normalized within total tissue-derived cells. Statistically significant correlations are marked: *$P < 0.05$ (nominal), **$P < 0.05$ (Benjamini–Hochberg adjusted). Color scale represents the strength and direction of the correlation (red: positive, blue: negative).

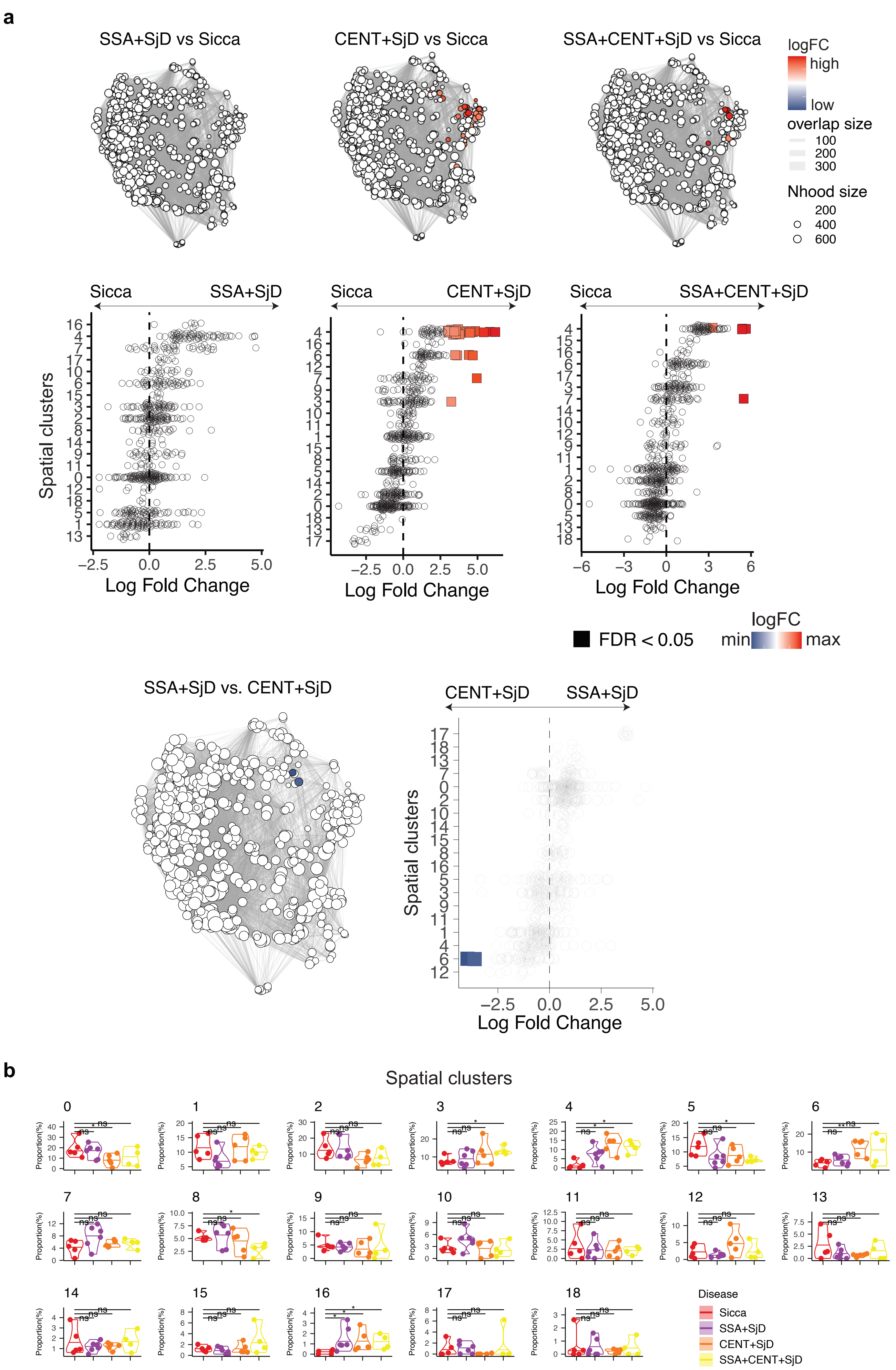
a
SSA+SjD vs Sicca
CENT+SjD vs Sicca
SSA+CENT+SjD vs Sicca
logFC
high
low
overlap size
100
200
300
Nhood size
200
400
600
Sicca
SSA+SjD
CENT+SjD
SSA+CENT+SjD
Spatial clusters
Log Fold Change
FDR < 0.05
logFC
min
max
SSA+SjD vs. CENT+SjD
b
Spatial clusters
Proportion(%)
Disease
Sicca
SSA+SjD
CENT+SjD
SSA+CENT+SjD

Supplementary Figure 7: Differential abundance profiles across spatial clusters in SjD subtypes.

a, Neighborhood graph of spatial regions and Beeswarm plots showing the distribution of $\log_2$-fold change (logFC) in neighborhoods in different spatial clusters in salivary glands. Colors indicate the logFC between case and controls. Neighborhoods that increased in case are shown in red. **b**, Proportion of spatial clusters per individual by autoantibody status.

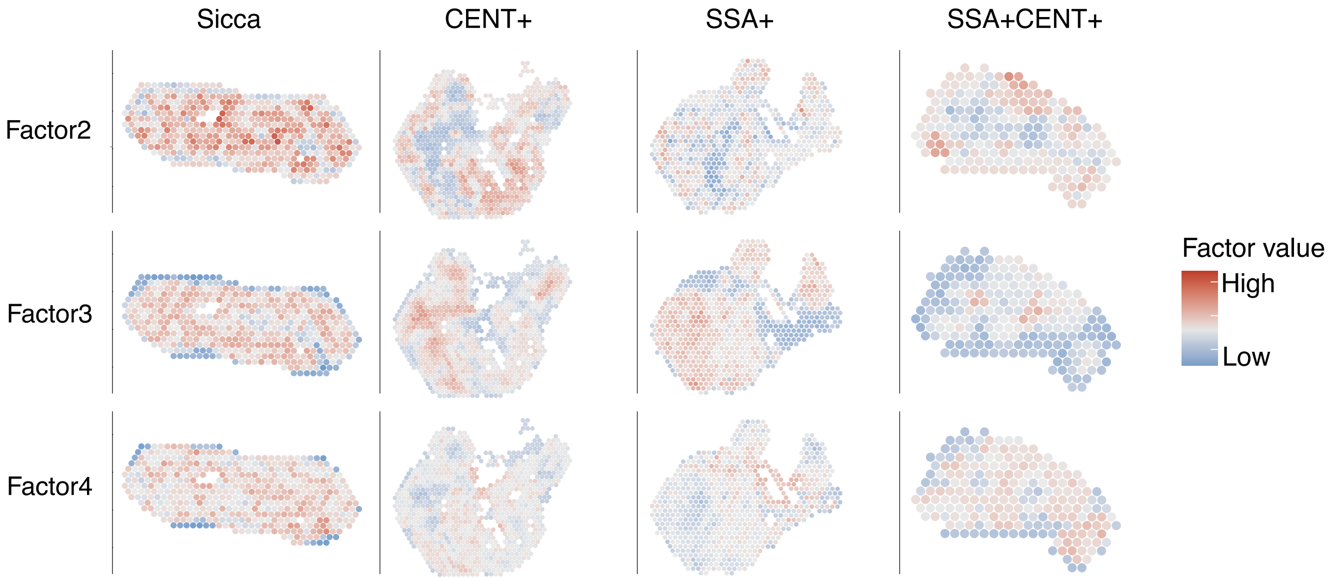

Sicca
CENT+
SSA+
SSA+CENT+
Factor2
Factor3
Factor4
Factor value
High
Low

Supplementary Figure 8: Spatial mapping of the factor values (Factor 2-4) of representative individuals with Sicca, CENT+, SSA+, and SSA+CENT+.

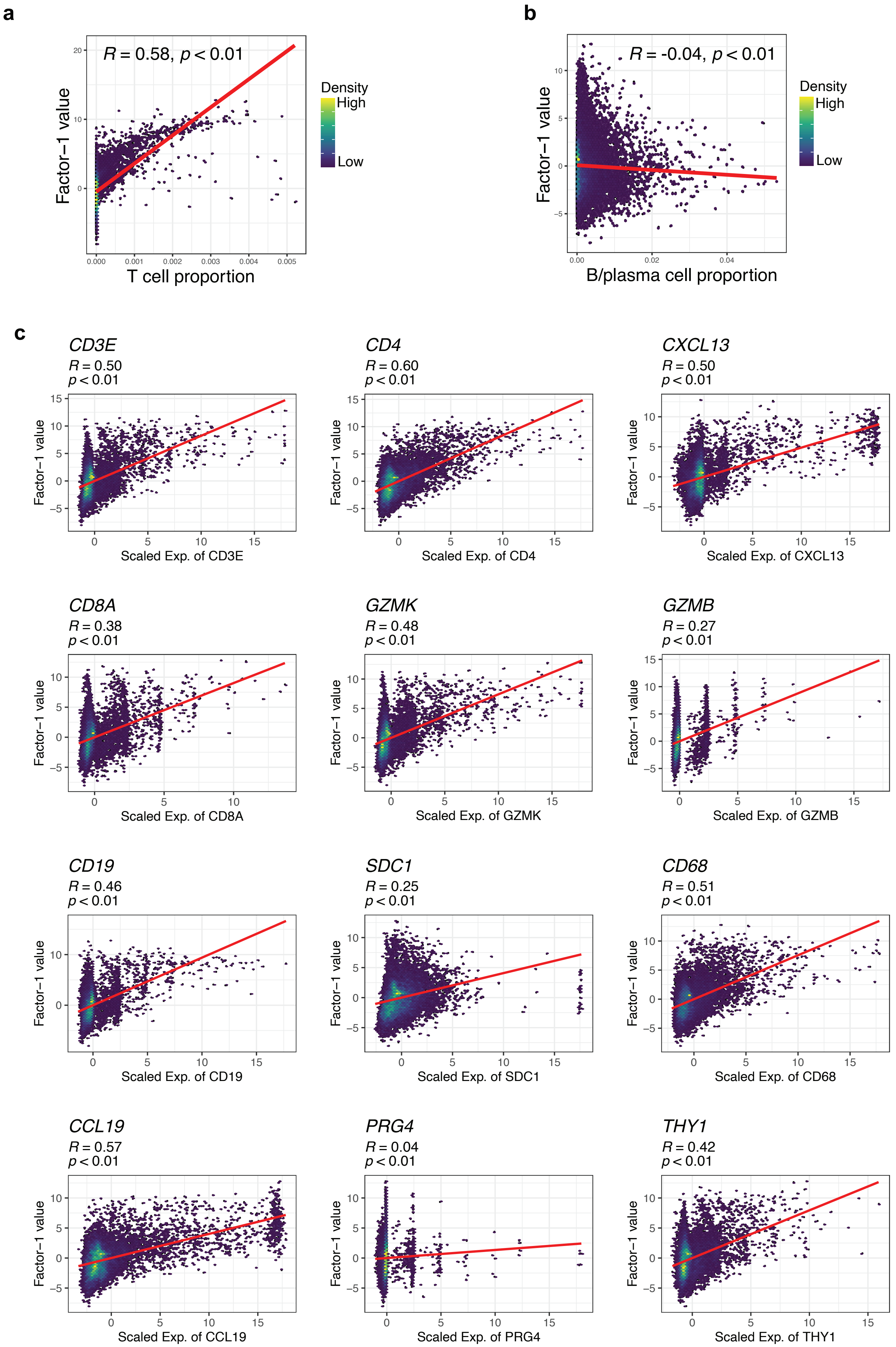
a
R = 0.58, p < 0.01
Factor−1 value
T cell proportion
0.000
0.001
0.002
0.003
0.004
0.005
Density
High
Low
b
R = -0.04, p < 0.01
Factor−1 value
B/plasma cell proportion
0.00
0.02
0.04
Density
High
Low
c
CD3E
R = 0.50
p < 0.01
Scaled Exp. of CD3E
CD4
R = 0.60
p < 0.01
Scaled Exp. of CD4
CXCL13
R = 0.50
p < 0.01
Scaled Exp. of CXCL13
CD8A
R = 0.38
p < 0.01
Scaled Exp. of CD8A
GZMK
R = 0.48
p < 0.01
Scaled Exp. of GZMK
GZMB
R = 0.27
p < 0.01
Scaled Exp. of GZMB
CD19
R = 0.46
p < 0.01
Scaled Exp. of CD19
SDC1
R = 0.25
p < 0.01
Scaled Exp. of SDC1
CD68
R = 0.51
p < 0.01
Scaled Exp. of CD68
CCL19
R = 0.57
p < 0.01
Scaled Exp. of CCL19
PRG4
R = 0.04
p < 0.01
Scaled Exp. of PRG4
THY1
R = 0.42
p < 0.01
Scaled Exp. of THY1
Factor−1 value

Supplementary Figure 9: Validation of MEFISTO Factor 1 as a marker of spatial immune activation.

a, Correlation between Factor 1 scores and the proportion of T cells in each Visium spot, as estimated by spatial transcriptomics deconvolution using the Redeconve pipeline with our scRNA-seq data as reference. **b**, Correlation between Factor 1 scores and the proportion of B/plasma cells per spot. **c**, Correlation plots between Factor 1 scores and expression levels of canonical marker genes for various cell types and immune pathways. Genes shown include markers for T cells (*CD3E, CD4, CD8A*), cytotoxic programs (*GZMK, GZMB*), B cells and plasma cells (*CD19, SDC1*), myeloid cells (*CD68*), fibroblasts (*PRG4, THY1*), and key immune chemokines (*CXCL13, CCL19*).

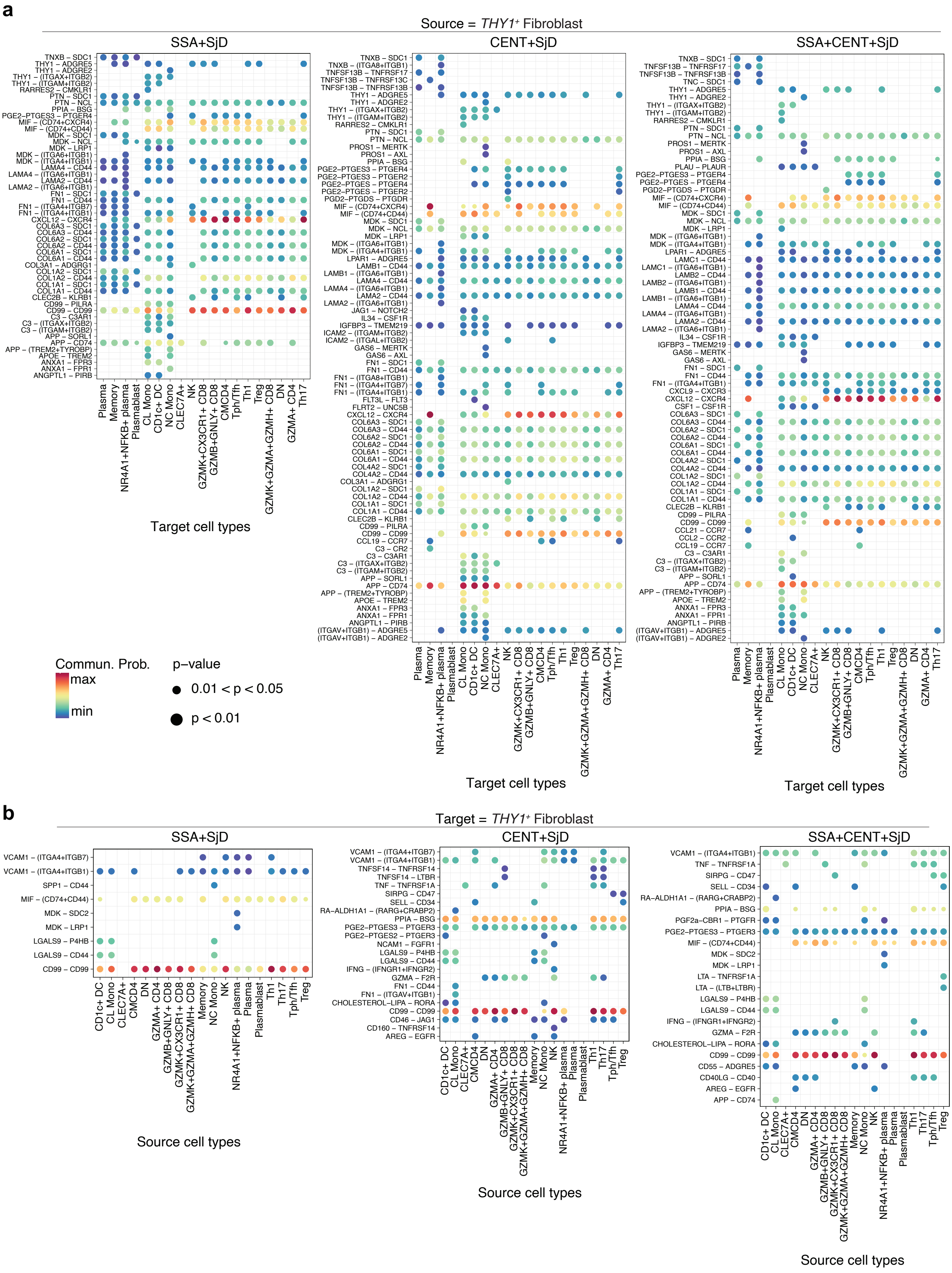
a
Source = THY1+ Fibroblast
SSA+SjD
CENT+SjD
SSA+CENT+SjD
Target cell types
Plasma
Memory
NR4A1+NFKB+ plasma
Plasmablast
CL Mono
CD1c+ DC
NC Mono
CLEC7A+
NK
GZMK+CX3CR1+ CD8
GZMB+GNLY+ CD8
CMCD4
Tph/Tfh
Th1
Treg
GZMK+GZMA+GZMH+ CD8
DN
GZMA+ CD4
Th17
Commun. Prob.
max
min
p–value
0.01 < p < 0.05
p < 0.01
b
Target = THY1+ Fibroblast
SSA+SjD
CENT+SjD
SSA+CENT+SjD
VCAM1 – (ITGA4+ITGB7)
VCAM1 – (ITGA4+ITGB1)
SPP1 – CD44
MIF – (CD74+CD44)
MDK – SDC2
MDK – LRP1
LGALS9 – P4HB
LGALS9 – CD44
CD99 – CD99
Source cell types
CD1c+ DC
CL Mono
CLEC7A+
CMCD4
DN
GZMA+ CD4
GZMB+GNLY+ CD8
GZMK+CX3CR1+ CD8
GZMK+GZMA+GZMH+ CD8
Memory
NC Mono
NK
NR4A1+NFKB+ plasma
Plasma
Plasmablast
Th1
Th17
Tph/Tfh
Treg

Supplementary Figure 10: Cell–cell communication analysis highlights the central role of *THY1*⁺ fibroblasts in SjD.

a-b, Heatmaps depicting predicted ligand–receptor interactions in which *THY1+* fibroblasts are either the signal-sending (**a**) or signal-receiving (**b**) population within each subgroup. Rows list the individual ligand–receptor pairs, while columns represent the interacting cell clusters. Color intensity indicates the communication probability. The size/shape of the dots denotes statistical significance.
