# Supplementary material for: Comparative single-cell and spatial profiling of anti-SSA-positive and anti-centromere-positive Sjögren’s disease reveals common and distinct immune activation and fibroblast-mediated inflammation
Source: Nat Commun. 2025 Sep 22;16:8299. doi: 10.1038/s41467-025-63935-9 (PMC12454658; doi:10.1038/s41467-025-63935-9)
Supplement: Supplementary file 2 — Description of Additional Supplementary Files [file 41467_2025_63935_MOESM2_ESM.pdf]

Description of Additional Supplementary Files

Supplementary Data 1: Characteristics of enrolled subjects.

Supplementary Data 2: Differentially expressed genes (adjusted p-values < 0.05) using scRNA-seq data by autoantibody-subgroups.

Supplementary Data 3: Gene set enrichment analysis (adjusted p-values < 0.05) using scRNA-seq data by autoantibody-subgroups.

Supplementary Data 4: Differentially expressed genes (adjusted p-values < 0.05) using Visium data by autoantibody-subgroups.

Supplementary Data 5: List of positively correlated pathways with Factor-1 score.
