# Supplementary material for: Comparative single-cell and spatial profiling of anti-SSA-positive and anti-centromere-positive Sjögren’s disease reveals common and distinct immune activation and fibroblast-mediated inflammation
Source: Nat Commun. 2025 Sep 22;16:8299. doi: 10.1038/s41467-025-63935-9 (PMC12454658; doi:10.1038/s41467-025-63935-9)
Supplement: Supplementary file 8 — Reporting Summary [file 41467_2025_63935_MOESM8_ESM.pdf]

nature portfolio

Corresponding author(s): Jun Inamo, Masaru Takeshita

Last updated by author(s): Aug 8, 2025

Reporting Summary

Nature Portfolio wishes to improve the reproducibility of the work that we publish. This form provides structure for consistency and transparency in reporting. For further information on Nature Portfolio policies, see our Editorial Policies and the Editorial Policy Checklist.

Statistics

For all statistical analyses, confirm that the following items are present in the figure legend, table legend, main text, or Methods section.

n/a	Confirmed	
☐	☒	The exact sample size (n) for each experimental group/condition, given as a discrete number and unit of measurement
☐	☒	A statement on whether measurements were taken from distinct samples or whether the same sample was measured repeatedly
☐	☒	The statistical test(s) used AND whether they are one- or two-sided<br>*Only common tests should be described solely by name; describe more complex techniques in the Methods section.*
☐	☒	A description of all covariates tested
☐	☒	A description of any assumptions or corrections, such as tests of normality and adjustment for multiple comparisons
☐	☒	A full description of the statistical parameters including central tendency (e.g. means) or other basic estimates (e.g. regression coefficient) AND variation (e.g. standard deviation) or associated estimates of uncertainty (e.g. confidence intervals)
☐	☒	For null hypothesis testing, the test statistic (e.g. F, t, r) with confidence intervals, effect sizes, degrees of freedom and P value noted<br>*Give P values as exact values whenever suitable.*
☒	☐	For Bayesian analysis, information on the choice of priors and Markov chain Monte Carlo settings
☒	☐	For hierarchical and complex designs, identification of the appropriate level for tests and full reporting of outcomes
☐	☒	Estimates of effect sizes (e.g. Cohen's d, Pearson's r), indicating how they were calculated

Our web collection on statistics for biologists contains articles on many of the points above.

Software and code

Policy information about availability of computer code

Data collection	No software was used for data collection.
Data analysis	We used publicly available software for the data analysis; CellRanger (v5.0.1), CellBender (v0.3.0), scDblFinder (v1.16.0), Seurat (v5.0.0), Harmony (v1.2.0), alakazam (v1.3.0), scRepertoire (v1.12.0), miloR (v1.20.0), Redeconve (v1.1.2), MOFA2 (v1.13.0), clusterProfiler (v4.10.0), and msigdbr (v7.5.1). Source code is available at GitHub (https://github.com/juninamo/Keio_SjS_singlecell/). Default parameters were used unless otherwise stated in the Method section.

For manuscripts utilizing custom algorithms or software that are central to the research but not yet described in published literature, software must be made available to editors and reviewers. We strongly encourage code deposition in a community repository (e.g. GitHub). See the Nature Portfolio guidelines for submitting code & software for further information.

Data

Policy information about availability of data

All manuscripts must include a data availability statement. This statement should provide the following information, where applicable:

- Accession codes, unique identifiers, or web links for publicly available datasets
- A description of any restrictions on data availability
- For clinical datasets or third party data, please ensure that the statement adheres to our policy

All raw and processed data have been deposited in the DNA Data Bank of Japan (DDBJ) via the National Bioscience Database Center (NBDC) Human Database under accession code JGAS000773.

Research involving human participants, their data, or biological material

Policy information about studies with human participants or human data. See also policy information about sex, gender (identity/presentation), and sexual orientation and race, ethnicity and racism.

Reporting on sex and gender	The study collected data on the biological sex of participants (male or female) as indicated in the Supplementary Table. Sex was considered in the study design and analysis to determine if findings differed by sex. No information on gender identity was collected. Sex and gender data has been reported where relevant in the manuscript.
Reporting on race, ethnicity, or other socially relevant groupings	All participants were Japanese individuals recruited from a single medical center in Japan. No other socially constructed or socially relevant categorization variables such as race or ethnicity were used in the analyses. Clear definitions of the relevant terms used and the method(s) used to classify people into the different categories are provided.
Population characteristics	The study included Japanese patients with Sjögren's disease who met the 2016 ACR/EULAR classification criteria and seronegative Japanese control subjects with xerostomia. Key characteristics including age, sex, disease duration, clinical parameters, and treatment status are provided in the Supplementary Table.
Recruitment	Sjögren's disease patients were recruited from Keio University Hospital between October 2016 and October 2022. Patients had not received glucocorticoids or immunosuppressants in the 3 months prior to sampling except for one patient stated in Methods section . Eight seronegative xerostomia subjects were also recruited as controls. All participants provided written informed consent.
Ethics oversight	The study protocol was approved by the medical ethics committee of Keio University Hospital and followed the Declaration of Helsinki principles. Informed consent was obtained from all participants.

Note that full information on the approval of the study protocol must also be provided in the manuscript.

Field-specific reporting

Please select the one below that is the best fit for your research. If you are not sure, read the appropriate sections before making your selection.

[x] Life sciences [ ] Behavioural & social sciences [ ] Ecological, evolutionary & environmental sciences

For a reference copy of the document with all sections, see nature.com/documents/nr-reporting-summary-flat.pdf

Life sciences study design

All studies must disclose on these points even when the disclosure is negative.

Sample size	The scope of our datasets and the methodological choices made were strategically aligned with our study's objectives and the current state of technological and financial feasibility.
Data exclusions	No data was excluded.
Replication	We analyzed multiple individuals with each autoantibody status to minimize inter-individual variability and validated our findings using multi-modal single-cell technologies.
Randomization	Randomization of the experimental design does not apply for our analysis; The nature of our study inherently limited the applicability of randomization in our experimental design.
Blinding	Blinding of the experimental design does not apply for our analysis; The nature of our study inherently limited the applicability of blinding in our experimental design.

Reporting for specific materials, systems and methods

We require information from authors about some types of materials, experimental systems and methods used in many studies. Here, indicate whether each material, system or method listed is relevant to your study. If you are not sure if a list item applies to your research, read the appropriate section before selecting a response.

Materials & experimental systems

n/a	Involved in the study
☐	☒ Antibodies
☐	☒ Eukaryotic cell lines
☒	☐ Palaeontology and archaeology
☒	☐ Animals and other organisms
☒	☐ Clinical data
☒	☐ Dual use research of concern
☒	☐ Plants

Methods

n/a	Involved in the study
☒	☐ ChIP-seq
☐	☒ Flow cytometry
☒	☐ MRI-based neuroimaging

Antibodies

Antibodies used

All antibodies used are listed in Methods section.
• APC anti-human CD45: BioLegend, Cat# 304012, Clone HI30, Lot# B272156, Dilution 1:200
• PE anti-human CD326 (EpCAM): BioLegend, Cat# 324205, Clone 9C4, Lot# B222942, Dilution 1:200
• MUC7: NOVUS, Cat# NBP2-50391, Clone 4D2-1D7, Lot# CRT/14/08, Dilution 1:10000
• PIP: NOVUS, Cat# NBP2-53226, Clone PIP/1571, Lot# 5304-1P190827, Dilution 1:400
• Cytokeratin 17: NOVUS, Cat# NBP2-44427, Clone KRT17/778, Lot# 3872-2P160617, Dilution 1:500
• Cytokeratin 5: Abcam, Cat# ab52635, Clone EP1601Y, Lot# GR3198825-2, Dilution 1:100
• CEACAM6: Santa Cruz, Cat# sc-59899, Clone 9A6, Lot# J1716, Dilution 1:100
• AGR2: Santa Cruz, Cat# sc-101211, Clone 6C5, Lot# G1019, Dilution 1:200
• Cytokeratin 14: Santa Cruz, Cat# sc-53253, Clone LL001, Lot# C2218, Dilution 1:200
• Caveolin-1: Santa Cruz, Cat# sc-70516, Clone 4H312, Lot# H2119, Dilution 1:200
• Cytokeratin 19: R&D Systems, Cat# MAB3506, Clone BA17, Lot# XG1031809A, Dilution 1:5000
• TFCP2L1 (LBP9): GeneTex, Cat# GTX31477, Polyclonal, Lot# 821903659, Dilution 1:5000
• Actin, Smooth Muscle Ab-1: Thermo, Cat# MS-113-R7, Clone 1A4, Lot# 113R 1208C, Dilution 1:1
• Goat anti-Mouse IgG1, Alexa Fluor 488: Thermo, Cat# A21131, Polyclonal, Lot# 73D1-1, Dilution 1:500
• Goat anti-Mouse IgG2b, Alexa Fluor 488: Thermo, Cat# A21141, Polyclonal, Lot# 1256170, Dilution 1:500
• Goat anti-Rabbit IgG (H+L), Alexa Fluor Plus 488: Thermo, Cat# A32731, Polyclonal, Lot# SC243838, Dilution 1:500
• Goat anti-Mouse IgG1, Alexa Fluor 546: Thermo, Cat# A21123, Polyclonal, Lot# 1249015, Dilution 1:500
• Goat anti-Mouse IgG2b, Alexa Fluor 546: Thermo, Cat# A21143, Polyclonal, Lot# 1711516, Dilution 1:500
• F(ab')2-Goat anti-Rabbit IgG (H+L), Alexa Fluor 546: Thermo, Cat# A11071, Polyclonal, Lot# 1322319, Dilution 1:500
• Rabbit anti-Human C3d Complement: DAKO, Cat# A0063, Polyclonal, Lot# 41602720, Dilution 1:200
• CD90 (Thy1): BioLegend, Cat# 328101, Clone 5E10, Lot# B349305, Dilution 1:100
• Anti-Human IgG, Fcγ Alexa Fluor 647: Jackson ImmunoResearch, Cat# 109-606-170, Polyclonal, Lot# 138919, Dilution 1:800
• Human IgG1 isotype control: BioXcell, Cat# BP0297, Polyclonal, Lot# 786120S1, Dilution: same as tested antibody (negative control)

Validation

Every antibody used in this study had been previously validated by the manufacturer.

Eukaryotic cell lines

Policy information about cell lines and Sex and Gender in Research

Cell line source(s)	Expi293 cells were purchased from Thermo Fisher Scientific. 293T cells were sourced from RIKEN CELL BANK.
Authentication	Cell line authentication was performed by the supplier.
Mycoplasma contamination	Cell lines used in this study were verified to be free of mycoplasma contamination by the supplier.
Commonly misidentified lines (See ICLAC register)	No commonly misidentified cell lines were used in this study.

Plants

Seed stocks: N/A

Novel plant genotypes: N/A

Authentication: N/A

Flow Cytometry

Plots

Confirm that:

- [x] The axis labels state the marker and fluorochrome used (e.g. CD4-FITC).
- [x] The axis scales are clearly visible. Include numbers along axes only for bottom left plot of group (a 'group' is an analysis of identical markers).
- [x] All plots are contour plots with outliers or pseudocolor plots.
- [x] A numerical value for number of cells or percentage (with statistics) is provided.

Methodology

Sample preparation: Sample preparations are described in Methods.

Instrument: All flow data were acquired on a FACS Aria Ⅲ (BD Biosciences).

Software: Analyses were carried out with FlowJo software (BD Biosciences).

Cell population abundance: These are properly described in the relevant figures.

Gating strategy: These are properly described in the relevant figures.

- [x] Tick this box to confirm that a figure exemplifying the gating strategy is provided in the Supplementary Information.
